# A Mask R-CNN-Based Approach for Brain Aneurysm Detection and Segmentation from TOF-MRA Data

**DOI:** 10.3390/brainsci15121295

**Published:** 2025-11-30

**Authors:** Emre Aykaç, Gürol Göksungur, Güneş Seda Albayrak, Mehmet Emin Yüksel

**Affiliations:** 1 Department of Biomedical Engineering, Graduate School of Natural and Applied Sciences, Erciyes University, 38039 Kayseri, Türkiye; emre.aykac@bozok.edu.tr; 2 Vocational School of Health Services, Yozgat Bozok University, 66100 Yozgat, Türkiye; 3 Department of Radiology, Yozgat City Hospital, 66100 Yozgat, Türkiye; gurolgoksungur@hotmail.com; 4 Department of Neurology, Yozgat Bozok University, 66100 Yozgat, Türkiye; drgunesalbayrak@yahoo.com; 5 Department of Biomedical Engineering, Faculty of Engineering, Erciyes University, 38039 Kayseri, Türkiye

**Keywords:** intracranial aneurysm, deep learning, Mask R-CNN, magnetic resonance angiography, image segmentation, artificial intelligence, health informatics, medical imaging

## Abstract

*Background:* Accurate detection of intracranial aneurysms, especially those smaller than 3 mm, remains a critical challenge in neurovascular imaging due to their subtle morphology and low contrast in Time-of-Flight Magnetic Resonance Angiography (TOF-MRA) scans. This study presents a Mask R-CNN-based deep learning framework designed to automatically detect and segment intracranial aneurysms, with specific architectural modifications aimed at improving sensitivity to small lesions. *Method:* A dataset of 447 TOF-MRA volumes (161 aneurysmal, 286 healthy) was used, with patient-level deduplication and 5-fold cross-validation to ensure robust evaluation. Bayesian hyperparameter optimization was applied using Optuna, and two key innovations were introduced: a Small Object Aware ROI Head to better capture micro-aneurysms and customized anchor configurations to improve region proposal quality. Healthy scans were incorporated as negative samples to enhance background modeling, and targeted data augmentation increased model generalization. *Results:* The proposed model achieved a Dice coefficient of 0.8832, precision of 0.9404, and sensitivity (recall) of 0.8677, with consistent performance across aneurysm sizes. *Conclusions:* These results demonstrate that the integration of architectural innovations, automated optimization, and negative-sample modeling enables a clinically viable deep learning tool that could serve as a reliable second-reader system for assisting radiologists in intracranial aneurysm detection.

## 1. Introduction

Brain aneurysms are balloon-like structures that develop due to weaknesses in the walls of cerebral arteries and may lead to serious consequences such as subarachnoid hemorrhage (SAH) in case of rupture [1]. Mortality rates can reach up to 50%, and a significant proportion of survivors suffer from permanent neurological damage [2]. Therefore, early and accurate diagnosis is of critical importance for appropriate treatment planning [3]. Time-of-Flight Magnetic Resonance Angiography (TOF-MRA) is widely used in the screening of intracranial aneurysms, as it does not require contrast agents and provides high contrast resolution [4].

However, manual evaluation of MRA images involves reviewing a large number of slices one by one, which increases the likelihood of missing small aneurysms or those located near bifurcation points [5]. For this reason, automatic and semi-automatic analysis methods can accelerate the diagnostic process, reduce the workload of radiologists, and assist in detecting lesions that might otherwise be overlooked [6].

Traditional image processing methods rely on techniques such as thresholding, edge detection, region growing, and morphological filtering [7]. While these approaches have been foundational in early computer-aided detection (CAD) systems, they fall short in complex anatomical regions such as cerebral vasculature. Specifically, arterial bifurcations, overlapping vessels, and intensity inhomogeneity in time-of-flight magnetic resonance angiography (TOF-MRA) images pose significant challenges. Consequently, such methods often yield a high rate of false positives (FPs) and struggle to reliably detect small aneurysms—typically less than 5 mm in diameter—which are of critical clinical relevance [8].

To overcome these limitations, deep learning-based approaches, particularly convolutional neural networks (CNNs), have been increasingly employed in medical image analysis. Numerous studies have demonstrated that deep learning models not only outperform traditional image processing methods in segmentation tasks but also offer better generalization and classification capabilities across diverse datasets and imaging modalities [9,10]. In the context of aneurysm detection and segmentation, CNN-based architectures have shown promising results, particularly when trained with large, well-annotated datasets. Fully segmentation-focused architectures such as U-Net [11] and V-Net [12] have been widely adopted in medical imaging applications due to their ability to learn rich contextual features at multiple scales. However, these models perform only segmentation and lack the inherent capability to localize and classify multiple distinct objects—an essential requirement when dealing with multiple aneurysms in a single MRA or CTA volume. In contrast, hybrid architectures that combine object detection and segmentation functionalities, such as Mask R-CNN, are well-suited for this task.

Mask R-CNN, proposed by He et al. [13] extends the object detection framework of Faster R-CNN by adding a parallel branch for predicting segmentation masks [14]. This enables simultaneous bounding box regression, classification, and pixel-level mask generation for each detected region of interest (ROI). In neurovascular imaging tasks, this architecture offers a critical advantage: it can precisely delineate the boundary of each aneurysm while also discriminating between true aneurysms and anatomical variants such as vascular loops or bifurcations. Dai et al. [15] used a Faster R-CNN (region proposal CNN) on CTA maximum-intensity-projection images to detect cerebral aneurysms (311 patients, 352 aneurysms). This 2D slice-based approach achieved 91.8% sensitivity overall, and 96.7% sensitivity for aneurysms >3 mm. The model performed consistently across aneurysm locations and processed each 3D case in  25 s, suggesting practicality for screening CTA [15]. Hwang et al. [16] employed Mask R-CNN (with custom loss functions) to detect and segment intraluminal thrombi in Abdominal Aortic Aneurysms (AAA) on CTA. Using 60 CTA volumes, their method achieved an F1-score of  0.92 for thrombus detection and a high Dice coefficient (0.83) for segmentation. This demonstrates Mask R-CNN’s ability to delineate vascular abnormalities in 3D scans with high accuracy, prioritizing minimal false negatives [16].

In recent years, applications of deep learning in time-of-flight magnetic resonance angiography (TOF-MRA) have demonstrated the potential of automated aneurysm detection systems, particularly in achieving high sensitivity. Ueda et al. [17] employed a patch-based 2D ResNet-18 CNN and reported a sensitivity of approximately 93%. However, neither Dice nor F1 scores were reported, limiting the interpretability of segmentation performance. Stember et al. [18], using a 2D U-Net on MIP images, achieved 98.8% sensitivity, but did not report metrics such as Dice or F1 scores, as all test cases were aneurysm-positive. Sichtermann et al. [19] applied a 3D convolutional neural network based on the DeepMedic architecture to TOF-MRA data comprising 85 cases with 115 annotated aneurysms. Their model achieved an overall sensitivity of 90%, increasing to 96% for aneurysms between 3–7 mm and 100% for aneurysms larger than 7 mm. The study emphasized the importance of pre- and post-processing to enhance model performance. Joo et al. [20] introduced a 3D ResNet model trained on 468 MRA exams, achieving 87.1% sensitivity and 92.0% specificity on the internal test set, along with 85.7% sensitivity and 98.0% specificity on an external dataset. Joo et al. [21] further validated their deep learning model in a retrospective, single-center, confirmatory clinical trial of 332 MRA exams, reporting 91.1% sensitivity and 93.9% specificity at the patient level, and 92.3% sensitivity at the lesion level. Sohn et al. [22] evaluated the clinical benefit of AI-assisted reading in a physician study, observing improved sensitivity—from 73.5% to 86.5%—across all readers, particularly among non-experts, supporting the model’s utility in assisting diagnostic accuracy in clinical settings.

To further improve detection performance, Terasaki et al. [23] proposed a multidimensional CNN (MD-CNN) that integrates both 2D and 3D information. Evaluated on a three-center dataset comprising 559 TOF-MRA scans, the MD-CNN achieved 89% sensitivity on external validation sets, showing better performance compared to conventional 2D and 3D CNNs. Di Noto et al. [24] introduced a weakly supervised 3D CNN that utilized coarse annotations and anatomical priors to reduce labeling effort. Their model reached 83% sensitivity on internal data and 68% on the external MICCAI ADAM dataset, highlighting challenges in generalization across different institutions and scanners.

In the field of computed tomography angiography (CTA), Park et al. [25] developed HeadXNet, a 3D U-Net model that increased radiologists’ sensitivity from 83.1% to 89.0% in a reader study, without affecting specificity or reading time. Similarly, Shi et al. [26] trained a 3D CNN on over 1000 CTA scans and achieved 97.5% sensitivity on internal data, maintaining strong performance during external validation. Their model modestly improved radiologist accuracy, demonstrating the benefit of AI assistance in detecting subtle lesions.

More recently, Bizjak et al. [27] proposed a cross-modal pipeline involving a two-step approach: vessel segmentation using nnU-Net, followed by aneurysm classification on vessel surfaces via PointNet++. Evaluated on over 3200 scans (1054 MRA, 2174 CTA), the model achieved 90% sensitivity on CTA and 85% on MRA. Sensitivity remained high even for aneurysms smaller than 3 mm, emphasizing its robustness and clinical applicability across imaging modalities.

A comparative overview of the most recent deep learning-based studies on intracranial aneurysm detection using TOF-MRA is presented in Table 1. This summary highlights variations in dataset composition, modeling approaches, and performance metrics across the literature, providing context for the effectiveness of the proposed model in this study.

In many previous studies, segmentation performance has primarily been reported in terms of sensitivity, which reflects the model’s ability to correctly identify true aneurysmal cases. This metric is conceptually equivalent to recall in classification terminology, as both measure the proportion of true positives correctly detected. However, sensitivity alone does not account for false positives and may therefore be insufficient, particularly in datasets with class imbalance or in clinical decision support settings where high precision is crucial. For this reason, our study reports not only recall but also precision—which penalizes false positives—along with the F1 score, which balances precision and recall, and the Dice Similarity Coefficient (DSC), which evaluates the spatial accuracy of segmentation. This comprehensive evaluation aims to capture not only the sensitivity of the model but also its accuracy and consistency in a more balanced and clinically meaningful manner.

Given these promising developments, the present study aims to evaluate the performance of a Mask R-CNN model trained on 3D TOF-MRA images using optimized hyperparameters via Optuna [28]. Particular attention is given to the ability of the model to segment aneurysms of varying sizes, with quantitative evaluation metrics such as Dice Similarity Coefficient (DSC), Precision, Recall, and F1-score being computed separately for small, medium, and large aneurysms.

To the best of our knowledge, no prior study has explored the application of a Mask R-CNN architecture with a ResNet-50 backbone for intracranial aneurysm detection using TOF-MRA data. This model was selected for its proven capability to perform simultaneous object detection and instance-level segmentation, which is particularly advantageous in cases with multiple or anatomically ambiguous aneurysms. Furthermore, the ResNet-50 backbone offers a favorable trade-off between model depth and computational cost, facilitating robust feature extraction even in complex vascular structures. Although most prior work has focused on segmentation-only architectures or CTA-based datasets, this study demonstrates that Mask R-CNN, when appropriately adapted to 2D slices of TOF-MRA volumes, can effectively localize and delineate aneurysms with competitive performance. Based on these considerations, our primary objective was not to develop a full 3D classification framework, but rather to design a sequence-based, pixel-level approach capable of distinguishing aneurysmal vessels from normal vasculature and accurately segmenting these regions. In particular, we aimed to build a decision-support system that can reliably detect small aneurysms—those most likely to be overlooked in routine clinical interpretation—by leveraging fine-grained 2D representations of TOF-MRA slices.

Mask R-CNN, with its ResNet-50 backbone, brings a two-stage detection-and-segmentation framework to aneurysm tasks. This is unusual in this domain: reviews consistently report that most aneurysm-detection pipelines use encoder–decoder CNNs (especially U-Net variants) for segmentation [29]. By contrast, Mask R-CNN first proposes candidate regions (like a generic object detector) and then refines each into an instance mask. Alternative architectures have been explored: vision–transformer hybrids (e.g., TransUNet) capture global context, and graph convolutional nets have been applied to vascular graphs for artery labeling, but these address different problems (classification or structural labeling rather than pixelwise aneurysm masks). Classical approaches typically involve explicit vessel segmentation via thresholding or model fitting, followed by rule-based lesion detection.

In parallel, recent work has emphasized that deep learning models for neurovascular imaging should provide spatially interpretable outputs and follow rigorous, attention-driven neuroimaging methodology [30,31]. Although our framework does not introduce an explicit attention module, the two-stage, region-proposal-based design of Mask R-CNN naturally focuses computation on a limited set of salient vascular regions and produces pixel-level aneurysm masks. These masks offer anatomically grounded visual evidence that radiologists can inspect alongside the original TOF-MRA images, which is consistent with this attention-focused, interpretable neuroimaging perspective.

Architecturally, Mask R-CNN’s combination of a Region Proposal Network (RPN) and ROIAlign is the key advantage: it treats each aneurysm as a distinct instance, which can help in cases with multiple nearby aneurysms. In practice, Mask R-CNN has shown competitive accuracy. For example, one comparative study reported that Mask R-CNN achieved a detection mAP of approximately 0.82 at IoU=0.5 on an aneurysm-like task, slightly better than a U-Net baseline (mAP ≈0.79) and nearly matching a TransUNet. However, this came with a cost: Mask R-CNN ran substantially slower (about 30 frames/s) than a pure U-Net (about 42 frames/s) on the same hardware. In other words, Mask R-CNN can yield high accuracy but has higher computational complexity. Thus, the novelty of our approach lies in adapting this powerful instance-segmentation model to TOF-MRA aneurysm data. Ultimately, its use is justified only if it provides tangible gains over well-tuned CNN or transformer baselines; current evidence suggests that it can be competitive in accuracy, but any improvements must outweigh its extra computational overhead [32].

To improve the model’s ability to generalize across different patient data, 5-fold cross-validation was implemented. This approach partitions the dataset into five equally sized subsets (folds), ensuring that each subset is used as a testing set exactly once while the remaining four serve as the training set. Five folds were chosen as a practical compromise between computational efficiency and statistical robustness. Using fewer folds (e.g., 2 or 3) may result in high variance in validation performance, whereas a higher number (e.g., 10) increases training time substantially without a proportionate gain in reliability, particularly in medical datasets where data preprocessing and model training are time-consuming.

For hyperparameter tuning, a Bayesian optimization strategy was adopted using the Optuna framework. This allowed for an efficient exploration of the hyperparameter space, including learning rate, batch size, number of iterations, and decision thresholds, all of which were selected based on validation set performance.

To enhance robustness against image variability, a range of data augmentation techniques were applied to the training data. These included geometric transformations such as rotation, as well as photometric augmentations like brightness and contrast adjustments and the addition of slight Gaussian noise. It is necessary to emphasize that horizontal and vertical flipping were not employed, as preserving anatomical orientation is critical in cerebral imaging tasks.

In addition to the base architecture, two targeted architectural modifications are introduced to improve the detection of small intracranial aneurysms (<3 mm). First, a custom Small Object Aware ROI Head is integrated into the Mask R-CNN framework. This specialized module processes low-area proposals separately using adjusted classification thresholds and, when needed, low-level FPN features to better preserve fine structural details. Secondly, the Region Proposal Network (RPN) is modified with a refined anchor box configuration, including smaller anchor sizes and broader aspect ratios, alongside optimized IoU thresholds. These enhancements significantly improve the sensitivity of the model and segmentation accuracy for small and hard-to-detect aneurysms.

## 2. Material and Methods

### 2.1. Dataset

In this study, two different data sources consisting of Time-of-Flight Magnetic Resonance Angiography (TOF-MRA) images were used for the detection and segmentation of intracranial aneurysms. A total of 161 aneurysmal cases were formed by combining 157 TOF-MRA sequences with aneurysms obtained from the OpenNeuro platform and 4 aneurysmal sequences obtained from Yozgat City Hospital. In addition, 286 healthy TOF-MRA images from Yozgat City Hospital were included in the model as background data for training the Mask R-CNN model.

All images were anonymized, and patient identifiers were removed. The TOF-MRA sequences obtained from Yozgat City Hospital were acquired on a Siemens MAGNETOM Amira 1.5T MRI scanner and were approved by the Ethics Committee of the Yozgat Provincial Directorate of Health and the Ethics Committee of Yozgat Bozok University.

All images obtained from the OpenNeuro platform (dataset: ds003949) were collected between 2010 and 2015 and were approved by the relevant Regional Ethics Committee. The OpenNeuro dataset consists of TOF-MRA scans acquired on a Siemens MAGNETOM Verio 3T MRI scanner, and all data were anonymized according to the Brain Imaging Data Structure (BIDS) standard and publicly shared under the CC0 license [33].

### 2.2. Manual Segmentation with ITK-SNAP

**Figure 1:**
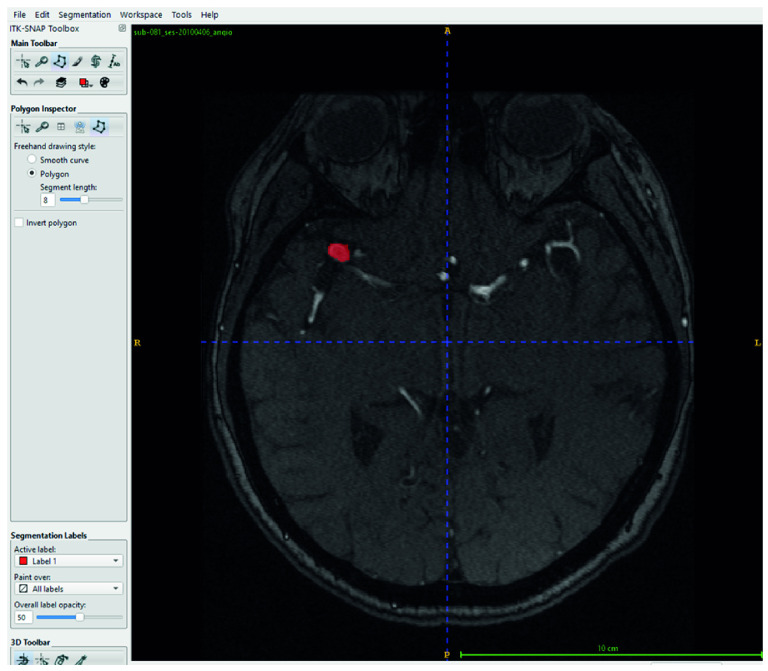
Manual segmentation of the aneurysm was performed using ITK-SNAP (version 4.2.0) on the axial view of the TOF-MRA scan. The aneurysm region was manually delineated by an expert radiologist using the Polygon Tool.

The segmentation of all aneurysmal cases used in this study was performed manually by an expert radiologist using the ITK-SNAP software version 4.2.0. During the segmentation process, each axial slice of the TOF-MRA images was carefully examined, and the boundaries of the aneurysms were manually delineated using the “Polygon Tool.” As a result of this process, accurately defined mask files were generated for each aneurysmal image as illustrated in Figure 1. Among the 290 TOF-MRA scans obtained from Yozgat City Hospital, 286 cases were labeled as “no aneurysm” after a brief screening. The remaining 4 aneurysmal cases were subjected to detailed segmentation and were manually masked. In addition, all 157 aneurysmal TOF-MRA cases obtained from the OpenNeuro platform were re-evaluated by the expert radiologist and manually re-segmented using ITK-SNAP. As a result of this process, a total of 161 aneurysmal and 286 healthy TOF-MRA images were prepared for use in the study.

### 2.3. Preprocessing

**Figure 2:**
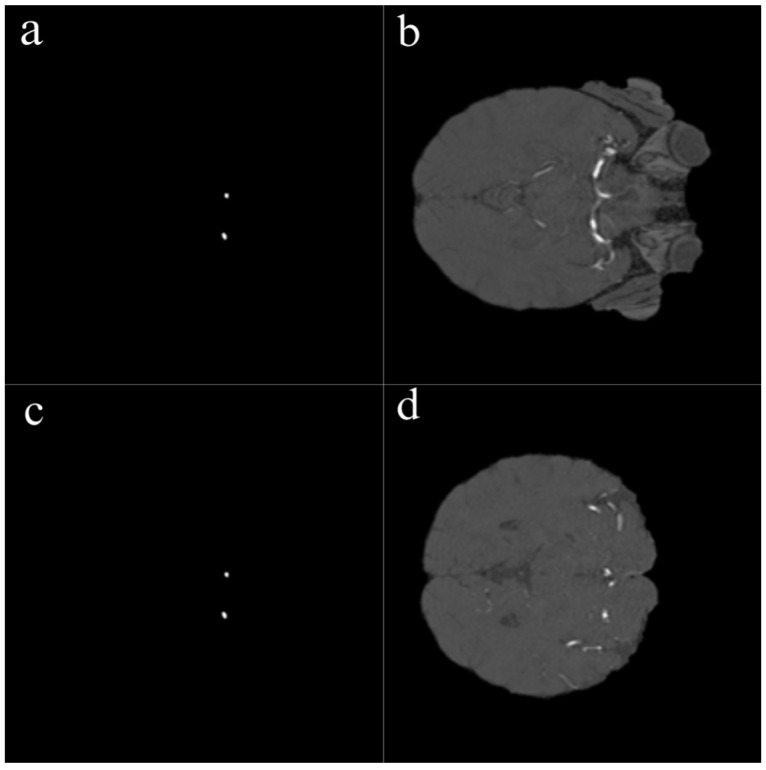
Sample 2D slices and corresponding aneurysm masks derived from TOF-MRA volumes. Left (**a**,**c**): Binary segmentation masks showing aneurysm regions in white. Right (**b**,**d**): Original TOF-MRA slices corresponding to each mask. Each 3D volume was converted into 2D axial slices, and manual annotations were used to generate ground truth segmentation masks.

In this study, several preprocessing steps were applied to prepare the TOF-MRA images for deep learning. To focus the model on intracranial structures, skull stripping was applied to all TOF-MRA images using FMRIB’s Brain Extraction Tool (BET). By adjusting parameters such as fractional intensity and surface inflation, brain tissue was effectively isolated. Processed images were manually reviewed, and necessary adjustments were made to ensure accurate brain extraction, resulting in a clean dataset suitable for segmentation. The raw data consisted of 3D volumetric brain images stored in NIfTI format (nii/nii.gz). To improve the training performance of the model and standardize the dataset, these volumes were converted into two-dimensional (2D) slices and saved in PNG format. Each image slice was then paired with its corresponding segmentation mask (Figure 2).

In the initial phase, the dataset was organized by creating training, validation, and test subsets. TOF-MRA images with and without aneurysms were separated into corresponding folders. To ensure the accuracy of segmentation procedures, the presence and correctness of mask files corresponding to each image were verified. Any missing or mismatched segmentation files were identified and either corrected or excluded from the dataset if necessary.

One of the most important steps in the preprocessing workflow was the conversion of 3D volumetric TOF-MRA images into two-dimensional slices. Each TOF-MRA sequence was sliced in the axial plane and converted into 2D images, with each slice saved as an individual image file. The same process was applied to the segmentation masks, ensuring that each aneurysmal region was paired with the corresponding 2D image.

To enable more efficient processing by the model, pixel intensity normalization was performed. This step aimed to reduce brightness variations across images, allowing for more consistent model learning. For each image, minimum and maximum pixel values were identified, and a rescaling to the range of 0–255 was applied. The normalization was carried out using the following formula:

(1) $$I_{\mathrm{norm}}=\mathrm{round}\left(\frac{I-I_{\min}}{I_{\max}-I_{\min}}\times 255\right)$$

where *I* is the original pixel intensity, $I_{\min}$ and $I_{\max}$ are the minimum and maximum pixel intensities in the image, respectively, and $I_{\mathrm{norm}}$ is the normalized pixel intensity.

In the final step of the preprocessing pipeline, all generated images and their corresponding segmentation masks were saved into designated directories and organized for use during model training. As a result, a processed and normalized dataset was created from the raw TOF-MRA data, making it suitable for training the deep learning model.

### 2.4. Data Augmentation

In this study, various data augmentation techniques were applied to enable better generalization of the deep learning model on TOF-MRA images. Expanding the dataset helps the model learn different variations, reduces the risk of overfitting, and improves its ability to detect small aneurysms.

The OpenCV library was used to perform image transformations and manipulations during the augmentation process. These transformations were applied not only to the images but also to their corresponding masks to maintain data consistency.

One of the primary techniques used was rotation. To increase the ability of the model to detect aneurysms from different angles, each image was rotated by a certain degree. Rotation was performed around the image center to avoid distortion of the original content and corresponding masks, and interpolation was used to minimize information loss. The rotation was applied using the following transformation matrix:

(2) $$M=\left[\begin{array}{ccc} \cos(\theta ) & -\sin(\theta ) & t_{x}\\ \sin(\theta ) & \cos(\theta ) & t_{y} \end{array}\right]$$

where θ is the rotation angle, and $t_{x}$, $t_{y}$ are the translation terms used to preserve the center of the image during rotation. Each image was rotated by $+15^{\circ }$ and $-15^{\circ }$ to improve the model’s ability to detect aneurysms from different orientations.

In addition to rotation, contrast adjustment was also applied. To enable the model to learn from variations in image brightness and contrast levels, contrast adjustment was applied. This process was performed by multiplying the pixel values of the image by a certain factor and modifying the brightness value. The contrast adjustment was applied using the following formula:

(3) $$I_{\mathrm{new}}=\alpha \cdot I+\beta$$

where $I_{\mathrm{new}}$ represents the adjusted image, α is the contrast factor, and β is the brightness value. In this study, the parameters were set as α=0.5 and β=−100 to reduce the dominance of high-intensity vessels and improve the visibility of low-contrast aneurysmal regions.

To further enhance visual features, gamma correction was utilized. This technique mimics the logarithmic response of the human eye and is commonly used to enhance details in low-contrast areas. The gamma correction was computed using the following formula:

(4) $$I_{\mathrm{new}}=255\times {\left(\frac{I}{255}\right)}^{1/\gamma }$$

where γ represents the gamma coefficient. In this study, γ=2.0 was used, which enhanced the visibility of the bright regions in the images.

### 2.5. Dataset Splitting and 5-Fold Cross-Validation

The dataset, consisting of NIfTI-format TOF-MRA images, was randomly shuffled and partitioned into five mutually exclusive folds for cross-validation. In each iteration, 80% of the data (four folds) was used for training, while the remaining 20% (one fold) served as an independent test set.

In total, 286 aneurysm-negative scans were used as background-only training data and were not included in the aneurysm-focused cross-validation analysis. The remaining 161 aneurysm-positive subjects, each comprising approximately 150–200 axial slices, were used to construct the 5-fold cross-validation splits. All images were annotated using subject-slice identifiers of the form sub_XX_slice_YY (e.g., sub_05_slice_115). By grouping on the subject prefix (sub_XX), we ensured that all slices from a given patient were always assigned to the same fold, so that no subject appeared in more than one split. Thus to avoid data leakage and ensure unbiased performance estimation, we strictly enforced patient-level separation between training and test folds, ensuring that no scans from the same patient appeared in both training and test sets.

Although training is performed on 2D slices with pixel-wise labels, all data splits were defined strictly at the patient level. Slices from the same 3D TOF-MRA volume share highly correlated anatomy, noise characteristics, and acquisition parameters; mixing such slices across training and test sets would lead to information leakage and artificially optimistic performance estimates. Therefore, each patient’s entire volume (all slices) was assigned exclusively to a single split (train or test), reflecting the realistic clinical scenario in which the model encounters completely unseen patients.

### 2.6. Mapping to 2D PNG Format

After converting the NIfTI-format images into 2D PNG format, the training and test datasets were updated accordingly. The original dataset.json files were used to map the NIfTI filenames to their corresponding PNG images, and new JSON files (dataset_fold.json) were generated for each fold. This resulted in five separate training and test sets in PNG format, prepared for cross-validation and model training.

### 2.7. Architectural Modifications for Small Aneurysm Detection

To enhance the sensitivity of Mask R-CNN for small aneurysms, several architectural modifications were introduced to improve the detection of lesions occupying very few pixels in TOF-MRA slices. Although clinical rupture-risk classifications typically define aneurysms smaller than 7 mm as “small” [34], this threshold does not reflect the actual detection difficulty in computer-vision-based segmentation. In practice, the greatest challenge arises for aneurysms smaller than 3 mm, which may appear in only one or two slices and cover a very limited pixel area. Therefore, consistent with prior work [24], we adopted a segmentation-oriented grouping: aneurysms <3 mm were considered small, those between 3–5 mm as medium, and those >5 mm as large. This grouping guided the design of architectural modifications aimed at improving sensitivity for the most pixel-sparse lesions.

During each forward pass, the physical area of every predicted region was computed in mm^2^ using voxel spacing values obtained directly from the NIfTI metadata, rather than assuming a fixed spacing. Empirically, we observed that very small bounding boxes were frequently suppressed by standard ROI operations (non-maximum suppression and score filtering). For typical voxel spacings in our dataset, this corresponded to regions with approximate areas below 25–35 mm^2^. Based on a series of pilot experiments, we selected 28 mm^2^ as the decision threshold for defining “small objects”: proposals below this area were treated as candidate small aneurysms.

For these small-object proposals, we applied a dynamic confidence-thresholding strategy to mitigate the bias of the ROI classifier toward larger structures. Concretely, the classification threshold was reduced for small regions (from 0.90 to 0.60), allowing plausible small aneurysm candidates to pass the classification stage instead of being prematurely discarded. In parallel, the ROI head was modified to become size-aware: small instances were processed with enhanced feature resolution by prioritizing lower FPN levels (P2–P3) and increasing the ROI pooling resolution (from the default to 14×14), thereby preserving fine vascular details.

Overall, the ROI module became explicitly size-aware through the combination of (i) physical size computation from image metadata, (ii) adaptive score filtering for small proposals, and (iii) high-resolution feature pooling for tiny regions. In our experiments, the 28 mm^2^ threshold provided the best trade-off: lower thresholds led to a noticeable increase in false positives, whereas higher thresholds caused systematic suppression of small aneurysm candidates during NMS and ROI scoring. With the chosen configuration, the model’s recall for small aneurysms improved, while performance for medium and large lesions remained stable.

### 2.8. Training Process and Hyperparameter Optimization

In this study, a deep learning model based on the Mask R-CNN architecture was trained to perform automatic detection and segmentation of intracranial aneurysms using TOF-MRA images. The Detectron2 framework was employed for implementation, offering a modular and flexible environment for object detection and instance segmentation tasks [35]. The training pipeline involved several sequential stages, including dataset preparation, annotation formatting, model configuration, hyperparameter tuning, and evaluation.

To ensure compatibility with Detectron2, all preprocessed images and their corresponding segmentation masks were converted into the COCO (Common Objects in Context) annotation format. Each aneurysmal image slice was associated with a manually created binary mask, which was represented in the COCO file using polygon-based contour information. In contrast, healthy TOF-MRA images were incorporated into the dataset without any annotation entries. These background-only samples, lacking annotated regions, were explicitly included to help the model distinguish normal vascular anatomy from pathological aneurysmal structures. By exposing the model to such negative examples, false positive detections were significantly reduced, particularly in areas where normal vessels might resemble aneurysms in structure or intensity.

Following the dataset formatting process, two separate COCO annotation files were generated for each fold: one for training and one for validation. Each file included both aneurysmal and non-aneurysmal cases, allowing the model to learn from a balanced distribution of pathological and normal anatomical patterns. The segmentation task was defined as single-class, focusing solely on the “aneurysm” category.

**Figure 3:**
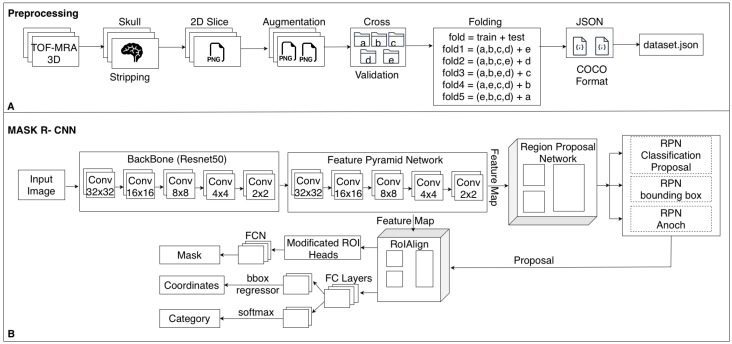
Overview of the proposed aneurysm detection and segmentation pipeline. (**A**) Preprocessing workflow. (**B**) Architecture of the adopted Mask R-CNN model.

The base model configuration was adopted from Detectron2’s standard mask_rcnn_R_50_FPN_3x.yaml, which employs a ResNet-50 backbone in combination with a Feature Pyramid Network (FPN). This configuration was customized to accommodate the specific characteristics of medical imaging data. Pretrained ImageNet weights were used to initialize the model, facilitating transfer learning and ensuring more stable convergence during training. The model was trained using Detectron2’s DefaultTrainer with GPU acceleration enabled on a workstation equipped with an NVIDIA RTX 3080 GPU running Ubuntu Linux. An overview of the complete preprocessing pipeline and the adopted Mask R-CNN architecture is summarized in Figure 3.

To enhance detection accuracy, especially for small aneurysms that are often challenging to localize, an extensive hyperparameter optimization process was conducted using the Optuna framework. Bayesian optimization was applied to explore the parameter space efficiently. The objective was to maximize validation performance in terms of Dice score and F1-score across all folds. Key parameters such as learning rate, batch size, number of iterations, region proposal thresholds, and anchor sizes were dynamically tuned. The optimization process was repeated for each fold in a 5-fold cross-validation setting to ensure robust and generalizable results.

Incorporating healthy TOF-MRA scans as negative training examples, along with fine-tuned hyperparameters and model adaptations, significantly improved both the sensitivity of the model. This strategy contributed to reducing false alarms while maintaining high detection performance, particularly in identifying small and morphologically variable aneurysms.

## 3. Results

### 3.1. Quantitative Evaluation

The segmentation performance of the model was assessed using 5-fold cross-validation. For each fold, precision, recall, F1 score, and Dice Similarity Coefficient (DSC) were computed based on the model’s ability to correctly identify aneurysmal regions. As summarized in Table 2, the model consistently achieved high segmentation performance across all folds, with mean precision of 90.29%, mean recall of 86.86%, and mean F1 and Dice scores of 88.32%.

**Figure 4:**
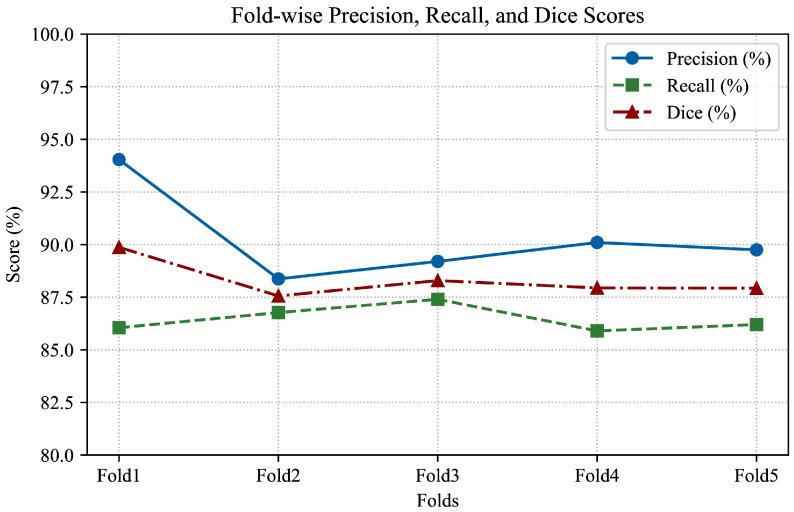
Fold-wise variation in model performance metrics.

The figure below illustrates the variation in Dice scores across different folds. Figure 4 shows that while Dice scores remained consistently high in the first four folds, a slight performance drop was observed in the fifth fold. This variation may reflect the influence of data distribution on model performance and highlights the importance of maintaining balanced and diverse datasets during cross-validation.

### 3.2. Performance by Aneurysm Size

**Figure 5:**
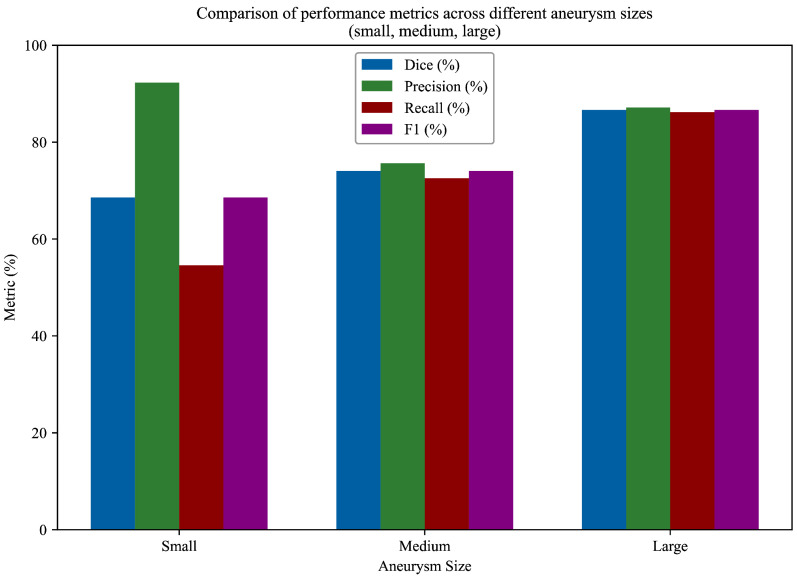
Comparison of performance metrics across different aneurysm sizes (small, medium, large).

**Figure 6:**
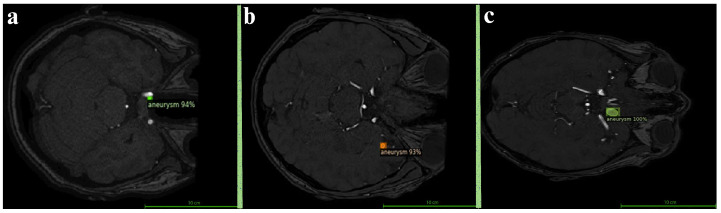
Sample model predictions based on aneurysm size. (**a**) Small aneurysm with 94% confidence (**b**) Medium aneurysm with 93% confidence (**c**) Large aneurysm with 100% confidence.

Figure 5 summarizes the Dice, precision, and recall scores for each size category for visualizing the variation in performance across aneurysm sizes. This comparison highlights the model’s robustness in detecting larger aneurysms while also pointing to areas for improvement, particularly in enhancing recall for smaller lesions. In order to better illustrate the model’s detection performance, Figure 6 presents representative segmentation results for small, medium, and large aneurysms. These qualitative examples demonstrate the ability of the model to recognize diverse morphological patterns—from subtle, low-intensity lesions to large, well-defined aneurysmal regions. A more detailed quantitative evaluation across different aneurysm sizes is provided in Table 3. The model achieved the highest Dice score for large aneurysms (86.67%), followed by medium (74.05%) and small aneurysms (68.57%). Notably, the model maintained high precision for small aneurysms (92.31%), suggesting strong discrimination capability and a low false-positive rate in these challenging cases. However, the relatively lower recall (54.55%) indicates difficulty in consistently capturing small aneurysms, which is a known limitation in the literature due to their low contrast and subtle appearance.

### 3.3. Best Hyperparameters

Hyperparameter optimization was conducted during Fold 1 training using the Optuna framework, with the aim of maximizing segmentation performance across key evaluation metrics. The selected configuration, summarized in Table 4, reflects a combination of automated Bayesian search and manual refinement, yielding a set of parameters that collectively improved model robustness, particularly in minimizing false positives without sacrificing sensitivity. A relatively low learning rate of 0.00110 was adopted to ensure stable and gradual convergence, which is especially important when dealing with high-resolution 3D medical images and limited lesion counts. The learning schedule involved decay milestones at iterations 4000 and 5500, with a decay factor (gamma) of 0.1, enabling the model to fine-tune its performance during later training stages. The maximum number of iterations was set to 6212, allowing sufficient training while mitigating overfitting. Notably, a high detection threshold (0.9457) was used during inference to suppress low-confidence predictions, resulting in a significant reduction of false positives. Although stricter thresholding can slightly reduce recall, it helps enhance clinical reliability by avoiding excessive false alarms. The batch size per image was set to 2, and the number of region proposals used for training the ROI head was increased to 256. These settings allowed the model to sample a wider variety of positive and negative instances, supporting better generalization. The Region Proposal Network (RPN) was further tuned with 512 proposals per image and a balanced positive fraction of 0.5, facilitating a more diverse learning of both aneurysmal and non-aneurysmal patterns. To improve localization performance, particularly for small aneurysms, the anchor configuration was adjusted to include five anchor sizes (8, 16, 32, 64, 128) and three aspect ratios (0.5, 1.0, 1.5). These values ensured appropriate coverage across varying aneurysm sizes and shapes. RPN IoU thresholds were set to 0.3 for foreground-background separation and 0.7 for positive anchor selection, while a moderate NMS threshold of 0.5203 was applied to retain relevant region proposals during inference.

The model was trained with Automatic Mixed Precision (AMP) and dynamic loss scaling, allowing efficient memory usage and faster computation, which is particularly beneficial in large-scale medical datasets. In addition, bitmask encoding was chosen for mask generation, providing pixel-level segmentation outputs that are better suited for precise delineation in medical imaging contexts such as TOF-MRA.

As outlined in Table 4, this carefully optimized hyperparameter setup significantly contributed to the overall performance of the model, balancing precision and recall while achieving high Dice scores. These refinements were especially beneficial for detecting small aneurysms and reducing false-positive detections, thereby supporting the development of a clinically reliable and generalizable deep learning framework.

## 4. Discussion

### 4.1. Key Advantages of Our Proposed Model

The proposed Mask R-CNN-based framework, optimized via Bayesian hyperparameter tuning, demonstrates a balanced and robust performance in the detection and segmentation of intracranial aneurysms using TOF-MRA data. The model achieved a precision of 94.0% and recall of 86.7%, positioning it among the first studies in the literature to simultaneously achieve such high performance on both axes in this domain. Unlike Ueda et al. [17], whose patch-based 2D CNN model attained high sensitivity (∼93%) but suffered from very low precision (estimated PPV ∼17–20%) due to excessive false positives, our model achieves a substantially better balance between sensitivity and precision. Similarly, while Stember et al. [18] and Sichtermann et al. [19] reported high sensitivity values (>90%), they did not provide comprehensive segmentation metrics such as Dice or F1-score, limiting comparative evaluation. In contrast, our model consistently reported a Dice score of 88.3% and F1-score of 88.3% across five folds, confirming its strong segmentation capability. Compared to Joo et al. [20] and Joo et al. [21], who used 3D ResNet models with specificity values between 92–98%, our approach achieves comparable accuracy while offering pixel-level segmentation masks—a significant advantage in clinical workflows where spatial localization and quantification are crucial. Despite the well-documented challenges of detecting aneurysms <3 mm in size, the proposed model achieved a Dice score of 68.57% for small lesions. While Bizjak et al. [27] reported a slightly higher sensitivity of 72% for small aneurysms, their study did not report segmentation-specific metrics such as Dice or F1-score. Thus, our approach provides a more granular and interpretable performance evaluation at the lesion level.

### 4.2. Impact of Using Healthy Cases as Background and Data Augmentation

In this study, instead of focusing solely on aneurysmal images, healthy TOF-MRA scans were also incorporated into the training process as background (negative) samples. This strategy allowed the model to learn not only the characteristics of aneurysmal regions but also the distribution of normal intracranial anatomy. Notably, this approach played a crucial role in reducing false positive (FP) rates, which is a common issue in many high-sensitivity models that lack exposure to sufficient negative samples. In our case, the inclusion of 286 healthy subjects significantly enhanced the model’s ability to differentiate true aneurysms from normal vascular structures.

As detailed by Qiao et al. [32], the performance of Mask R-CNN is strongly influenced by how well the background class is modeled: complex or under-represented backgrounds tend to generate spurious detections and reduce precision. In their PCB-monitoring study, Mask R-CNN frequently over-segmented normal regions, and the authors highlighted background variability and insufficient negative sampling as major sources of false positives, recommending richer background data to stabilize the classifier. By analogy, incorporating 286 healthy TOF-MRA scans into our training set provides abundant, anatomically diverse negative examples. Because Mask R-CNN explicitly includes a background class in its ROI classifier, every proposal in these healthy scans is forced to be labeled as background, sharpening the decision boundary between normal vasculature and aneurysmal tissue and thereby contributing to higher precision and lower FP rates.

The use of healthy cases also helps to mitigate the severe class imbalance characteristic of aneurysm segmentation, where aneurysm voxels occupy only a tiny fraction of the image. Guerrero et al. [36] showed that combining data-level rebalancing with loss-level adjustments (e.g., targeted augmentation of minority patterns together with weighted losses) substantially improves the classification of under-represented classes without degrading majority-class performance. Our strategy of adding a large pool of full-scan healthy controls plays an analogous role at the image level: it enriches the distribution of true-negative examples and constrains the network to focus on truly abnormal features rather than overfitting to aneurysm-like noise. In practice, this distribution-aware design regularizes the detector under strong foreground–background imbalance and supports the balanced sensitivity–precision trade-off observed in our results.

In addition, a series of data augmentation techniques were applied to improve the generalization ability of the model and reduce the risk of overfitting. Transformations such as rotation, brightness and contrast adjustment, and the addition of low-level Gaussian noise helped the model become more robust to anatomical and acquisition variability. These augmentations were especially useful for improving detection of small aneurysms under varying imaging conditions. Flipping operations were deliberately excluded, as the anatomical orientation of cerebral structures holds clinical significance and should be preserved.

Taken together, these two strategies—the use of healthy scans for background modeling and controlled data augmentation—contributed significantly to the model’s balanced performance. They enhanced the model’s reliability by reducing false detections while maintaining sensitivity across a wide range of anatomical and imaging variations.

### 4.3. Limitations

Despite the strong performance achieved by the proposed Mask R-CNN-based framework, several limitations must be acknowledged. First, the model operates on 2D TOF-MRA slices rather than full 3D volumetric data. Although this slice-wise strategy enabled fine-grained pixel-level segmentation and facilitated the detection of small aneurysms, it does not capture inter-slice continuity, which radiologists routinely consider during clinical evaluation. Future studies incorporating true 3D architectures or hybrid 2.5D representations may further improve contextual understanding and reduce ambiguity in borderline cases.

Second, subject-level specificity could not be reported because our evaluation framework is based on pixel-level instance segmentation rather than subject-level classification. In pixel-wise segmentation studies, metrics such as Dice, precision, and recall are computed over lesion masks rather than per-subject diagnostic outcomes, a practice also observed in several prior TOF-MRA segmentation works [18,19,24]. As a result, specificity—which requires true-negative subjects—cannot be meaningfully derived under a slice-wise segmentation paradigm. Instead, our analysis emphasizes lesion-level and pixel-level performance, which aligns with the objectives of instance segmentation and provides clinically relevant localization accuracy.

Third, although five-fold patient-level cross-validation was rigorously applied, the dataset originates from a limited number of clinical centers and includes scans acquired on both 1.5T and 3T MRI systems. While this heterogeneity improves generalizability, subtle scanner-related intensity differences may influence the model’s learned features. Multi-center datasets with standardized acquisition protocols would enable a more systematic analysis of scanner variability.

Finally, although architectural modifications such as the small-object-aware ROI head improved sensitivity for aneurysms smaller than 3 mm, the detection of ultra-small lesions remains challenging due to their extremely limited pixel footprint and occasional visibility in only one or two slices. Future work may integrate transformer-based global attention modules or multi-view consistency strategies to further mitigate this limitation.

### 4.4. Clinical Applicability

Although the current study does not yet constitute a clinically deployable system, the proposed framework demonstrates the capability to function as a preliminary computer-aided alerting tool. Because the 2D slice-level predictions can be programmatically merged into volume-level outputs using simple reconstruction steps, the model can highlight suspicious vascular regions across the full TOF-MRA study. In this form, the system has the potential to assist radiologists by drawing attention to subtle aneurysms—particularly small lesions that are more easily overlooked—while leaving the final diagnostic decision entirely to the clinician. Further work, including external validation and prospective reader studies, would be required before any clinical integration.

## 5. Conclusions

In this study, we developed a Mask R-CNN-based framework for the automated detection and segmentation of intracranial aneurysms on TOF-MRA images. By incorporating healthy cases as background samples, applying targeted data augmentation, and introducing architectural modifications that enhance small-lesion sensitivity, the model achieved strong performance with a Dice score of 0.883, precision of 0.94, and recall of 0.868. The proposed approach showed improved detection of aneurysms smaller than 3 mm, which are typically the most challenging in routine clinical assessment. These results demonstrate that a carefully adapted instance-segmentation model can provide accurate localization while maintaining high diagnostic reliability. Although further multi-center and prospective validation is required, the method shows clear potential as a supportive tool to assist radiologists in the early identification of intracranial aneurysms.

## Figures and Tables

**Table 1:** Comparison of recent studies on intracranial aneurysm detection.

Study (Year)	Dataset (Cases)	Method/Model	Performance Metrics
Ueda et al. (2019)	TOF-MRA (Train: 683, Test: 588)	Patch-based 2D ResNet-18 CNN	Sn: 91% (internal) Sp: –
Stember et al. (2019)	TOF-MRA MIP (Train: 250, Test: 86)	2D U-Net (MIP)	Sn: 98.8% AUC: 0.87
Sichtermann et al. (2019)	TOF-MRA (85 cases, 115 aneurysms)	3D CNN (DeepMedic)	Sn (overall): 90% Sn (3–7 mm): 96% Sn (>7 mm): 100%
Joo et al. (2020)	TOF-MRA (468 train, 170 test, 106 ext.)	3D ResNet CNN	Sn: 85.7–87.1% Sp: 92–98% PPV: 91.5–92.8%
Joo et al. (2021)	TOF-MRA (332 cases)	3D CNN	Sn (patient-level): 91.1% Sp: 93.9% Lesion Sn: 92.3%
Sohn et al. (2021)	TOF-MRA (332 cases)	DL-CAD	Reader Sn: 73.5% → 86.5% (with AI) Sp: –
Terasaki et al. (2022)	TOF-MRA (559 cases, 3 centers)	2D + 3D MD-CNN	Sn (internal): 89.4% Sn (external): 89.1%
Di Noto et al. (2023)	TOF-MRA (284 internal, 113 external)	Weakly-supervised 3D CNN	Sn (internal): 83% Sn (external): 68%
Bizjak et al. (2024)	1054 TOF-MRA + 2174 CTA	nnU-Net + PointNet++	MRA Sn: 85% (72% for <3 mm) CTA Sn: 90% (83% for <3 mm)
Park et al. (2019)	CTA (818 scans)	HeadXNet (3D U-Net)	Sn: 94.9% Radiologist Sn: 83.1% → 89.0% (with AI)
Shi et al. (2020)	CTA (1068 train, 400 external)	3D CNN + post-processing	Sn (internal): 94.4% Sn (external): 84.6% Lesion Sn: 76.1–84.1%
**This study (2025)**	TOF-MRA (447 total: 161 aneurysmal, 286 healthy)	**2D Mask R-CNN + Optuna**	**DSC: 88.32%** **Precision: 94.04%** **Sn (Recall): 86.77%**

**Legend:** Sn = Sensitivity; Sp = Specificity; PPV = Positive Predictive Value; AUC = Area Under Curve; DSC = Dice Similarity Coefficient; MIP = Maximum Intensity Projection; TOF-MRA = Time-of-Flight Magnetic Resonance Angiography; MRA = Magnetic Resonance Angiography; CTA = Computed Tomography Angiography; CNN = Convolutional Neural Network; DL-CAD = Deep-Learning Computer-Aided Detection; MD-CNN = Multi-Scale/Multi-Dimensional CNN; AI = Artificial Intelligence.

**Table 2:** Segmentation performance per fold.

Fold	Precision (%)	Recall (%)	F1 Score (%)	Dice (%)
1	94.04	86.05	89.87	89.87
2	88.37	86.77	87.56	87.56
3	89.20	87.40	88.29	88.29
4	90.10	85.90	87.94	87.94
5	89.75	86.20	87.93	87.93

**Table 3:** Performance by aneurysm size. In particular, the high precision for small aneurysms is noteworthy.

Aneurysm Size	Dice (%)	Precision (%)	Recall (%)
Small	68.57	92.31	54.55
Medium	74.05	75.62	72.55
Large	86.67	87.16	86.19

**Table 4:** Optimized hyperparameters obtained from Bayesian search and manual refinement for Fold 1 training.

Parameter	Value
Learning Rate (BASE_LR)	0.00110
Max Iterations (MAX_ITER)	6212
Batch Size (IMS_PER_BATCH)	2
ROI Batch Size (BATCH_SIZE_PER_IMAGE)	256
Score Threshold (SCORE_THRESH_TEST)	0.9457
RPN NMS Threshold (RPN.NMS_THRESH)	0.5203
Learning Rate Decay Steps	(4000, 5500)
Gamma (LR decay factor)	0.1
AMP Enabled	✓ (dynamic loss scaling)
Pretrained Weights	R-50 from ImageNet (Detectron2 Model Zoo)
Anchor Sizes	8, 16, 32, 64, 128
Anchor Aspect Ratios	0.5, 1.0, 1.5
RPN IoU Thresholds	0.3, 0.7
RPN Batch Size per Image	512
RPN Positive Fraction	0.5
Input Mask Format	bitmask

## Data Availability

The data used in this study are available from the OpenNeuro repository (dataset: ds003949) and from Yozgat City Hospital upon reasonable request. The publicly available dataset can be accessed at: https://openneuro.org/datasets/ds003949.

## Abbreviations

The following abbreviations are used in this manuscript:

MRA	Magnetic Resonance Angiography
TOF–MRA	Time-of-Flight Magnetic Resonance Angiography
CTA	Computed Tomography Angiography
MIP	Maximum Intensity Projection
DSC	Dice Similarity Coefficient
F1	F1-score
PPV	Positive Predictive Value
AUC	Area Under the Curve
IoU	Intersection over Union
NMS	Non-Maximum Suppression
FPN	Feature Pyramid Network
ROI	Region of Interest
RPN	Region Proposal Network
CNN	Convolutional Neural Network
AMP	Automatic Mixed Precision
COCO	Common Objects in Context
NIfTI	Neuroimaging Informatics Technology Initiative
BET	Brain Extraction Tool
BIDS	Brain Imaging Data Structure
DL–CAD	Deep Learning-based Computer-Aided Detection
MD–CNN	Multidimensional Convolutional Neural Network
